# Pharmacovigilance processes in low- and middle-income countries: moving from data collection to data analysis and interpretation

**DOI:** 10.1177/20420986241300006

**Published:** 2025-06-11

**Authors:** Olga Menang, Peter van Eeuwijk, Karen Maigetter, Andy Stergachis, Christian Burri

**Affiliations:** Department of Medicine, Swiss Tropical and Public Health Institute, Kreuzstrasse 2, Allschwil 4123, Switzerland; University of Basel, Basel, Switzerland; Department of Epidemiology and Public Health, Swiss Tropical and Public Health Institute, Allschwil, Switzerland; University of Basel, Basel, Switzerland; Department of Epidemiology and Public Health, Swiss Tropical and Public Health Institute, Allschwil, Switzerland; University of Basel, Basel, Switzerland; Department of Pharmacy, School of Pharmacy, University of Washington, Seattle, WA, USA; Department of Global Health, School of Public Health, University of Washington, Seattle, WA, USA; Department of Medicine, Swiss Tropical and Public Health Institute, Allschwil, Switzerland; University of Basel, Basel, Switzerland

**Keywords:** analysis, benefit-risk evaluation, data collection, decision-making, interpretation, low- and middle-income countries, pharmacovigilance, regulatory action, safety

## Abstract

**Background:**

The analysis and interpretation of pharmacovigilance data is an essential component of the continuous benefit-risk assessment of authorised medicinal products. Effective pharmacovigilance data analysis starts with data collection and involves critical activities, such as signal detection, that enable the generation of new information on marketed products, and inform safety-related regulatory actions. This real-time pharmacovigilance data analysis, which requires efficient collaboration and exchange of information between the key pharmacovigilance stakeholders, represents a challenge for many low- and middle-income countries (LMIC).

**Objectives:**

To assess the capacity for analysis of pharmacovigilance data in LMIC and to identify mechanisms to strengthen data analysis, interpretation and evidence-based pharmacovigilance decision-making.

**Design:**

We used a convergent parallel mixed-methods study design consisting of qualitative and quantitative methods.

**Methods:**

Qualitative and quantitative methods consisted of semi-structured interviews and an online survey, respectively. Quantitative research was complemented by cross-sectional analyses of the number of adverse event reports from LMIC in VigiBase^®^ from 2019 to 2023.

**Results:**

Nine key informants from eight countries were interviewed and 50 respondents from 34 countries completed the online survey. Four major themes emerged from the data and are proposed as transformative actions to strengthen pharmacovigilance data analysis and interpretation in LMIC: build on existing pharmacovigilance data analysis capacity rather than create new or parallel mechanisms; implement standardised procedures to enable efficient data analysis; augment the work of the safety committees by assigning pharmacovigilance staff to data analysis; and implement mechanisms that allow benefit-risk evaluation and decision-making.

**Conclusions:**

Findings from this research revealed that many LMIC have implemented procedures for reporting and collecting suspected adverse events, but a considerable proportion of the data collected is not analysed in-country due to a lack of requisite knowledge, processes and structures to support such analysis. Establishing the four essential elements proposed by this research will equip LMIC for efficient data analysis, thereby supporting consistent decision-making through pharmacovigilance.

## Plain language summary

Progressing from collecting information on side effects of medicines in developing countries, to analysing the information and using the results to guide decisions that promote the safe use of medical products

Why was the study done?

Analysing information on side effects of medicines is important for making sure that medicines are safe and effective. By doing so, it is possible to get new information on medicinal products, which can be used by authorities that approve medicine use to take actions that make medicines safer for the users. This has been successfully achieved in developed countries but for many developing countries the process is challenging. This research aims to understand how side effects are collected and analysed in developing countries and to suggest ways to improve this important activity.

What did the researchers do?

To understand the situation better, we interviewed experts from different developing countries and conducted an online survey. Nine experts from eight countries were interviewed and 50 people from 34 countries completed the online survey. We also assessed the number of side effects reports from developing countries in a global database from 2019 to 2023.

What did the researchers find?

Four major themes were identified and are proposed to help analyse and interpret side effects in developing countries: use the skills and systems that are already in place instead of starting new ones; have standard steps for analysing the information collected; adequately train staff to routinely analyse side effects; and find practical ways to turn the data into actions.

What do the findings mean?

This study shows that many developing countries are now able to collect information about adverse events of medicines, but they do not fully utilise the information they collect because they lack the knowledge and skill to analyse the information. If developing countries apply the four themes that are proposed in this study, they can strengthen the analysis of information on side effects and make more consistent decisions about medicine safety.

## Introduction

The analysis of pharmacovigilance (PV) data is indispensable for the continuous benefit-risk assessment of authorised medicinal products. Adverse drug reactions (ADR) are an important cause of morbidity and mortality, causing a huge burden for patients, health professionals and healthcare systems.^1–3^ Evidence of risks and benefits of medicines continue to emerge throughout the life-cycle of a medicinal product, especially information about rare ADR, ADR specific to sub-populations not included in clinical trials, ADR appearing after long-term use, medication errors, ineffectiveness, drug interactions, misuse, abuse and even unexpected therapeutic benefits. Detecting, evaluating, preventing and communicating these medicine-related issues necessitate a comprehensive functional PV system with the relevant structures and processes in place that enable continuous benefit-risk assessment of medicinal products throughout their life cycles.^4^

Continuous safety monitoring implies accurate and efficient collection and management of PV-related data. This involves several critical steps including the collection of suspected adverse events (AE) from all sources; causality assessment; identification of safety signals; risk management and mitigation; all of which enable the generation of new information on marketed products.^5,6^ The outcomes of these activities inform and drive regulatory decisions such as communication to key PV stakeholders, update of product information or safety labels, additional risk minimisation measures, product recalls, and in extreme circumstances, product withdrawal from the market.^7–9^ Achieving this PV maturity level requires complex interactions, collaborations and exchanges of information between key PV stakeholders such as national regulatory authorities (NRA) and related entities, for example, safety advisory bodies, marketing authorisation holders (MAH), healthcare professionals, patients and consumers, with the necessary structures and processes in place. This is a process established in developed countries with stringent regulatory requirements vis-à-vis PV. In the European Union (EU), for example, the European Medicines Agency provides detailed instructions on the execution of each critical PV process.

In low- and middle-income countries (LMIC), the situation is different. A majority of LMIC have yet to attain World Health Organisation (WHO) Global Benchmarking Tool maturity level (ML) 3 on a scale of 1–4, that is, a well-functioning and integrated regulatory system for medicines.^10^ A recent assessment of PV in LMIC showed that although a majority of countries have the legal and organisational framework for PV, essential PV activities were not implemented or performed, and many LMIC were unable to collect and utilise local data to identify signals and support regulatory decisions.^11^ The most reported impediments included low recorded ADR volumes, poor data quality, inadequate PV tools, insufficient stakeholder coordination and limited financial and human resources.^11–13^ PV strengthening initiatives in LMIC in the last decades have primarily focused on increasing the reporting of ADR.^14^ These efforts have borne their fruits, as shown by the increase in ADR reports from LMIC in VigiBase^®^, the WHO global database of AE reports for medicines and vaccines. In 2017, there were increases of 5%, 25% and 61% in individual case safety report (ICSR) contributions from Asian, African and Latin American countries, respectively, compared to 2016; in 2021 and 2022, there was a major surge due to the introduction of COVID-19 vaccines.^15,16^ The PV data collected is only valuable if it is adequately analysed and interpreted, with continuous benefit-risk evaluation to support decisions and actions that prevent ADR and promote public health. However, the majority of PV data and safety-driven regulatory actions in LMIC originate from actions taken in developed countries, rather than from local in-country data.

There are several reasons why it is imperative for LMIC NRA to be in a position to adequately analyse and interpret local PV data. Firstly, LMIC are introducing novel vaccines and medicines, some of which target diseases solely endemic in LMIC, for example, malaria and Ebola.^14^ Secondly, in the last decades, there has been a steady growth in pharmaceutical development and manufacturing in LMIC to meet local needs, and to reduce the cost of health products as well as dependency on developed markets.^17,18^ Thirdly, with new product launches exclusively in LMIC or simultaneously across the globe, for example, COVID-19 vaccines, LMIC regulators will not always be able to rely on the safety assessments conducted in developed countries, especially when local environmental and genetic influences play a role.^19–21^ Consequently, signal detection and benefit-risk evaluation will increasingly be required and occur in settings with limited PV capacity. The increased volume and complexity of marketed therapeutics and vaccines pose an increased concern for AE and highlight the need to accurately and efficiently evaluate local safety data.^22^

While previous assessments of PV capacity in LMIC using standardised tools described the capacity of PV systems, including data analysis capacity by the fulfilment of defined indicators,^11,23,24^ this research aims to assess the capacity of analysis of PV data in LMIC and to propose steps that enable progression from data collection to data analysis, interpretation and the application of the information for evidence-based decision-making in PV in LMIC.

## Methods

### Study design

We used a convergent parallel mixed-methods design, consisting of qualitative and quantitative methods. Qualitative and quantitative research consisted of semi-structured interviews and an online survey, respectively, focusing on the same thematic questions. In addition, a retrospective analysis of suspected AE in VigiBase^®^ was conducted, to understand trends in suspected AE reporting rates in LMIC and to determine whether the increase in volumes of AE reports was matched by a corresponding increase in data analysis.

### Sampling, setting and study population

Participants included representatives from the NRA, national immunisation programmes, academia, non-governmental organisations and global technical and donor agencies (hereafter referred to as technical and financial partners [TFP]). For the interviews, countries were selected based on the publicly available information on their PV maturity level (ML), such that every PV ML was adequately represented. Potential participants were contacted via email addresses provided by their organisations or via regional PV mailing lists. In addition, authors of articles that were included in a scoping review of strategies to build PV in LMIC written by the same authors were contacted directly by email.^14^ At least one LMIC from each WHO Region was identified.^25^ Sampling was purposive, based on informants’ knowledge and expertise in PV and decision-making position within the national or global PV organisations concerned. It was deemed sufficient to interview eight to twelve key informants, given that current knowledge suggests that saturation can be achieved in a narrow range of interviews, particularly in studies with relatively homogeneous study populations and narrowly defined objectives.^26^ The definite sample size was determined by the willingness of potential informants to participate in the research.

For the survey, the identification of countries and participants was the same as for the interview. The number of participants was estimated based on the number of LMIC who were full members of the WHO Programme for International Drug Monitoring at the start of the research, indicating that the minimum requirements for a functional PV system were present. The definite sample was determined by the willingness of potential informants to participate in the research. For this research, the definition of LMIC was according to World Bank Group country classifications.^27^ High-income countries (HIC) and key informants from HIC were excluded from the research.

### Data collection

Qualitative data were collected through semi-structured interviews. An interview guide (Supplemental File 1) was developed based on the study’s objective. This guide comprised 2 parts with 17 core questions: Part 1 consisted of key informant’s role in the national PV system and Part 2 investigated how LMIC national PV systems are moving from PV data collection to data analysis and interpretation. The interview guide was reviewed by the co-authors for clarity and validity of questions, and pilot-tested by a PV expert not directly involved in the research. Sixteen key informants were invited by email to participate in the interviews and were provided with an overview of the research. Verbal informed consent for the use of information provided in the research and for the interview recording was obtained at the start of each interview. The interviews were conducted via Zoom videoconferencing (Zoom Video Communications, Inc., San Jose, CA, USA) by the first author, from November 2021 to July 2022. The duration of the interviews ranged from 60 to 90 min.

Survey data were collected using a standardised questionnaire consisting of core 21 core questions made of 2 sections, similar to the interview guide (Supplemental File 2). The questionnaire was set up in ODK (https://getodk.org/) and pilot-tested by a PV expert not directly involved in the research. The questionnaire, in both English and French, was distributed by email and WhatsApp Messenger (WhatsApp Inc., San Diego, CA, USA) between November 2022 and February 2023. Three reminders were sent to non-respondents.

Two cross-sectional analyses of VigiBase^®^ were conducted to extract (1) the cumulative number of ADR per country on 9 August 2022 and (2) the cumulative number of ADR per WHO region from 1967 to 18 October 2023. Our analysis focused on data from 2019 to 2023, as this period was marked by the introduction of the first malaria vaccine (RTS, S/AS01-Mosquirix™) in 2019, and COVID-19 vaccines in 2021.

### Data analysis

The Framework Method was used for the thematic analysis of qualitative data.^28^ The respondents’ statements from the interviews were verbatim transcribed by the first author and a deductive approach was used to code the transcript. For this purpose, a codebook (Supplemental File 3) was developed with predefined codes, which were assigned to the transcribed data, line by line. Related subcodes were created to improve the accuracy of the analysis. The codes were clustered into categories and Microsoft Excel (Microsoft Corporation, 2016) was used to summarise and ‘chart’ each transcript into a matrix consisting of categories, codes and subcodes (Supplemental File 4). The data were interpreted, with the identification of patterns, relationships, differences and similarities leading to new thematic groups. Quantitative data collected in ODK were exported into Microsoft Excel 2016 for analysis. Data analysis consisted of descriptive statistics, primarily frequencies and percentages for categorical variables.

The Consolidated Criteria for Reporting Qualitative Research guidelines^29^ were consulted when preparing the manuscript (see Supplemental File 5 for the completed checklist).

## Results

### Characteristics of interview informants

Nine key informants from eight countries agreed to participate in the research and were interviewed (see Table 1(a)). Four persons did not respond to the invitation and the information collected from three key informants from HIC is not included in this assessment of data analysis capacity in LMIC. Seventy-eight percent (*n* = 7) were from sub-Saharan Africa (SSA) and 67% (*n* = 6) worked for an NRA or a National Pharmacovigilance Centre (NPVC). Participants’ PV experience averaged approximately 16 years (see Table 1(b)).

**Table 1.** (a) Countries participating in the interviews.

WHO region	Country	Count of respondents
African	Burkina Faso	1
	Côte d’Ivoire	1
	DRC	1
	Eritrea	1
	Ghana	1
	Malawi	2
Europe	Kazakhstan	1
South-East Asia	India	1

DRC, Democratic Republic of the Congo; WHO, World Health Organization.

**Table 1.** (b) Characteristics of interview informants.^a^

Characteristics	Number (%)
Organisation	
Academia	1 (11)
NIP	1 (11)
TFP	1 (11)
NPVC	2 (22)
NRA	4 (44)
Position	
National PV Focal Point	1 (11)
Pharmacist	1 (11)
Regulatory Officer	1 (11)
Chief Regulatory Officer	1 (11)
NRA Chairperson	1 (11)
PV Technical Officer/Senior Officer	2 (22)
Head of PV	2 (22)
Years of PV experience	
6–10	1 (11)
11–15	4 (44)
>16	4 (44)

a Reported positions rephrased and similar terms grouped for ease of presentation.

NIP, National Immunisation Programme; NPVC, National Pharmacovigilance Centre; NRA, National Regulatory Authority; PV, Pharmacovigilance; TFP, Technical and Financial Partner.

### Characteristics of survey respondents

The questionnaire was distributed to 81 persons and 50 respondents (62%) from 34 countries completed the questionnaire (see Table 2(a)). Participants’ PV experience ranged from less than 1 to over 16 years and 62% (*n* = 31) worked for the NRA and the NPVC (see Table 2(b)). Seventy-six percent (*n* = 38) of respondents were from SSA.

**Table 2.** (a) Countries participating in the survey.

WHO region	Country	Count of respondents
African	Angola	1
	Benin	1
	Burkina Faso	3
	Burundi	1
	Cameroon	2
	Central African Republic	1
	Congo	1
	Côte d’Ivoire	1
	DRC	3
	Gabon	1
	Guinea	1
	Guinea Bissau	1
	Kenya	1
	Liberia	1
	Malawi	2
	Mali	2
	Mauritania	1
	Mozambique	1
	Namibia	3
	Niger	1
	Nigeria	2
	São Tomé and Príncipe	1
	Sierra Leone	2
	Togo	1
	United Republic of Tanzania	2
	Zimbabwe	1
Americas	Brazil	3
	Mexico	1
Eastern Mediterranean	Morocco	1
Europe	Kazakhstan	1
South-East Asia	India	2
	Nepal	2
Western Pacific	Malaysia	1
	Philippines	1

DRC, Democratic Republic of the Congo; WHO, World Health Organization.

**Table 2.** (b) Characteristics of survey respondents.^a^

Characteristics	Number (%)
Organisation	
Other	1 (2)
Industry	2 (4)
Academia	5 (10)
NIP	5 (10)
TFP	6 (12)
NPVC	11 (22)
NRA	20 (40)
Position	
Communication Expert	1 (2)
Head of Department	1 (2)
National PV Commission Chairperson	1 (2)
PV Intern	1 (2)
PV Manager	1 (2)
Senior Lecturer	1 (2)
Medical Advisor	1 (2)
Not reported	1 (2)
Pharmaceutical Advisor	2 (4)
Professor Pharmacology	2 (4)
PV Coordinator	3 (6)
PV Technical Officer/Senior Officer	4 (8)
National PV Focal Point	4 (8)
NIP PV Focal Point	4 (8)
Regulatory Officer	5 (10)
Scientist/Senior Scientist	6 (12)
Head of PV	12 (24)
Years of PV experience	
<1	3 (6)
2–5	12 (23)
6–10	15 (30)
11–15	12 (23)
>16	8 (16)

a Reported positions rephrased and similar terms grouped for ease of presentation.

NIP, National Immunisation Programme; NPVC, National Pharmacovigilance Centre; NRA, National Regulatory Authority; PV, Pharmacovigilance; TFP, Technical and Financial Partner.

### Themes, categories and codes emerging from the qualitative data

Four major themes emerged from the qualitative research as shown in Table 3: (1) strengthen existing capacity for analysis of national PV data rather than create new or parallel mechanisms; (2) implement standardised procedures for efficient analysis of PV data; (3) augment the work of the national safety expert review committees (hereafter referred to as safety committees) by assigning PV staff to routine data analysis; and (4) implement mechanisms that enable benefit-risk evaluation and decision-making.

**Table 3.** Themes, categories and codes emerging from the qualitative data.

Themes	Categories	Codes
Strengthen existing capacity for analysis of national PV data	Status of PV data analysis	Data analysis trigger
		Capacity of data analysis
	Strengthening data analysis capacity	Gaps in PV data analysis
		Participants’ recommendations
Implement standardised procedures for efficient analysis of PV data	Guidelines for PV stakeholders	Guidelines for stakeholders, e.g., MAH and PHP
	SOP for analysis of PV data	SOP for critical PV processes
Augment the work of safety committees by assigning PV staff to routine data analysis	Data analysis responsibility	Safety Committee
		PV staff
	Functionality of the Safety Committee	Experience and empowerment
		Frequency of convening
	Analysis of PV data by PV staff	Experience of national PV staff
		Periodicity of assessment of other PV data
Implement mechanisms that enable benefit-risk evaluation and decision-making	Signal detection and benefit-risk evaluation	Periodicity of assessment of other PV data
		Signal detection
	Safety-driven regulatory actions	Recent regulatory actions
		Source of data

PV, Pharmacovigilance; SOP, Standard Operating Procedures; MAH, Marketing Authorisation Holders; PHP, Public Health Programmes.

The findings of the research are presented by categories below. Additional data collected from the quantitative research are rendered alongside the qualitative data.

#### Status of PV data analysis

Sixty-seven percent (six out of nine) of interview participants indicated that their countries had sufficient capacity for data analysis and all informants reported that staff were sufficiently competent. Three key informants reported inadequate data analysis due to a lack of procedures and structures. This was affirmed by a representative from a technical agency: ‘*Capacity is available, but data analysis is not done because logistics and procedures are missing*’ (TFP representative, LMIC).

**Figure 1.**
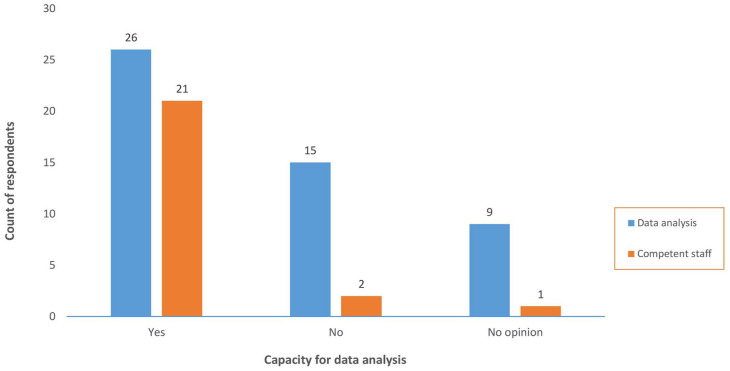
Capacity for data analysis versus availability of competent staff.

Quantitative data revealed that 26 (52%) participants from 17 countries agreed that the capacity for analysis of national PV data was adequate and 25 (50%) indicated that there were sufficiently competent staff assigned to perform vigilance activities. Of the 26 participants who agreed that the capacity for analysis of national PV data was adequate, 21 (81%) also affirmed that PV staff were skilled and competent (see Figure 1).

The main trigger for data analysis reported by interview informants was the introduction of new medicinal products especially vaccines and a corresponding increase in AE reported. Analysis of PV data was therefore the logical next step in the evolution of a PV system. Also with increased awareness of PV and collaboration with numerous stakeholders, questions arose on the outcome AE reported: ‘*We had many questions if PV was only for collecting data –* “*where are the fruits*” *– this triggered the need for data analysis, signal detection and communication*’ (NRA representative, LMIC).

#### Strengthening data analysis in LMIC

Major gaps in the analysis of PV data identified were the lack of procedures and tools, poor data quality, insufficient national coordination of PV activities, limited number of experienced personnel, overreliance on safety committees and the lack of country ownership of PV data, as it was reported by a representative of a technical agency: ‘*Lack of ownership of PV system and processes; many PV regulatory decisions are copied from developed countries*’ (TFP representative, LMIC). Participants’ recommendations to address these gaps included the following: establishing quality management systems with adequate and easily accessible tools and procedures; strengthening international collaboration and data sharing; continuous training to build PV staff expertise; facilitating information technology (IT) solutions such as data mining; and ensuring stable funding to sustain staff and functions of the safety committees.

#### Guidelines for PV stakeholders

All key informants affirmed that national PV guidelines were in existence. These guidelines mainly described the organisation of the national PV system and the roles and responsibilities of key PV stakeholders without providing details on how to carry out the defined tasks. The guidelines also describe reporting and collection of AE, and in some countries, for instance Ghana, there were guidelines for activities such as signal management, periodic safety update reports (PSUR), risk management plans (RMP) and safety communications: ‘*Yes, there are PV guidelines for PSUR, RMP, signal management, etc. We have PV templates and tools, and a tracker for signal management activities. Several signals have been detected and communicated*’ (NRA representative, LMIC).

#### Standard operating procedures for analysis of PV data

Key respondents from all countries affirmed that procedures for reporting and collection of AE were implemented and functional and there was a national safety database for management of AE. With the exception of Malawi and Côte d’Ivoire, key informants reported the availability of documented and implemented standard operating procedures (SOP) for critical activities such as signal management, periodic reporting and RMP: ‘*Yes, for each PV section, there are well documented processes; over 35 safety signals have been identified*’ (NRA representative, LMIC). For Malawi and Côte d’Ivoire, although procedures for major PV activities were not documented, activities such as reporting of AE and causality assessment were adequately described in PV guidelines or training materials, as affirmed by a representative from a technical agency: ‘*Many countries do not have SOP. PV activities are mainly described in training manuals. SOP are definitely needed and would be very helpful*’ (TFP representative, LMIC).

**Figure 2.**
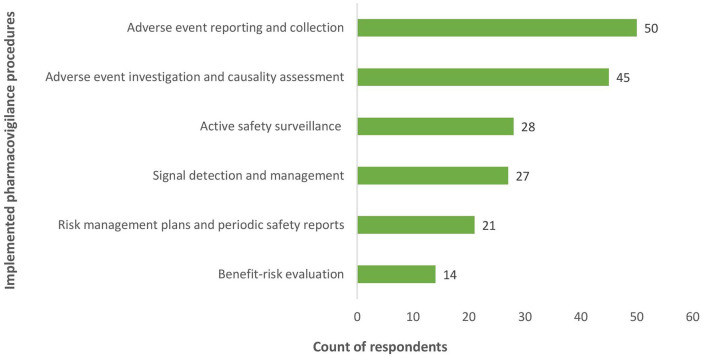
Implemented pharmacovigilance procedures.

From the quantitative research, all respondents (100%) affirmed that procedures for AE reporting and collection were implemented and functional, whereas only 14 (28%) reported that procedures for benefit-risk evaluation were implemented (see Figure 2).

#### Responsibility for analysis of national PV data

Qualitative findings showed that in all countries, except Kazakhstan, there was at least one safety committee, with the primary responsibility to assess causal relationships for serious adverse events (SAE), adverse events of special interest (AESI) and clusters of AE. The safety committees also contributed to the management of some complex AE (e.g. Democratic Republic of the Congo [DRC]) supported the signal review panel (e.g. Eritrea) and reviewed aggregate data and trends (e.g. Ghana). With the exception of Malawi, the PV staff were responsible for causality assessment of non-SAE. In some countries such as Ghana, DRC and Eritrea, the PV staff also carried out other tasks such as signal detection and aggregate data review: ‘*PV staff are very well trained and competent in PV. Only experienced staff perform data entry and also do medical assessment of case reports*’ (NPVC representative, LMIC).

**Figure 3.**
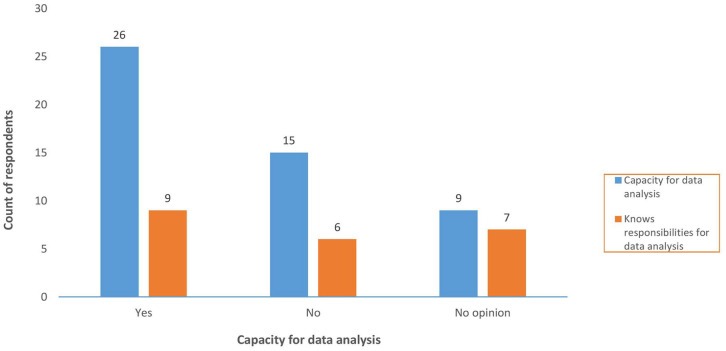
Responsibility for analysis of data that is not assessed by safety committees versus capacity for data analysis.

Quantitative findings revealed that less than 50% (*n* = 22) of respondents could identify who was responsible for analysing other PV data, that is, data that were not assessed by the safety committees. Of the 26 respondents who had indicated that there was adequate capacity to analyse PV data, only 9 could affirm that analysis of other PV data was done and could designate the entity responsible for the analysis (see Figure 3).

#### Functionality of safety committees

**Figure 4.**
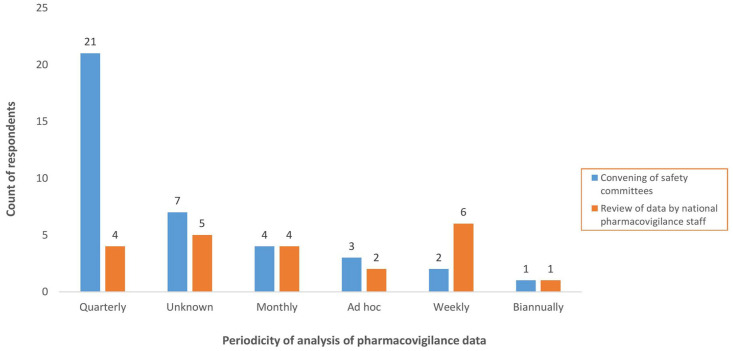
Periodicity of analysis of pharmacovigilance data.

There was at least one safety committee in each country which was functional, and operated based on the terms of reference proposed by the WHO, and used WHO tools for investigation and causality assessment of SAE: ‘*Safety committees are adequately trained and empowered to analyse PV data; however, all are not aware that their responsibility for data analysis goes beyond causality assessment*’ (TFP representative, LMIC). The safety committees convened at least quarterly, or more frequently, based on the needs (see Figure 4). The challenge for several countries was ensuring regular convening, and hence, the functionality of the safety committees because this required considerable financial and logistics mobilisation: ‘*The safety committee is functional; however, there are challenges for them performing their function such as regular meetings, continuous training, and site visits for investigations*’ (NRA representative, LMIC).

From the quantitative research, 38 (76%) respondents from 30 (83%) countries affirmed that there was a national safety committee, which reportedly convened quarterly for 21 of 38 respondents (55%) and the least frequent, biannually (3%), according to one respondent (see Figure 4).

#### PV data analysis by PV staff

A majority of interview respondents (six out of nine) indicated that PV staff were sufficiently trained and had the necessary expertise for data management and signal detection. Nevertheless, data were not systematically periodically analysed due to the challenges presented above, except in countries with clearly documented PV procedures: ‘*Data are reviewed regularly; every other month in preparation for committee meeting*’ (NRA representative, LMIC).

From the quantitative research, 24 (48%) of respondents indicated that PV staff had the required PV expertise to perform data analysis. The frequency of analysis of PV data by the PV staff is also shown in Figure 4.

#### PV data in a national safety database

**Figure 5.**
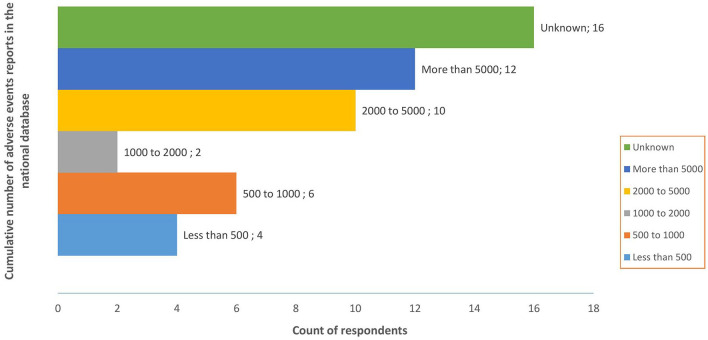
Number of adverse events in the national safety database.

Quantitative data showed that all countries had a PV database for the collation and handling of suspected AE. VigiBase^®^/VigiFlow^®^ (64% (*n* = 32)) and ODK (24% (*n* = 12)) were the databases reportedly used by a majority of countries. The number of AE in the safety database ranged from less than 500 to more than 5000 (see Figure 5).

**Figure 6.**
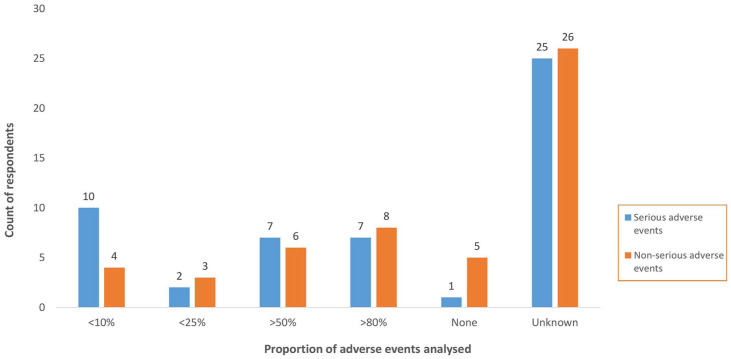
Proportion of serious and non-serious adverse events analysed.

Concerning the proportion of suspected AE in the safety databases that have been analysed, at least 50% (*n* = 25) of respondents did not know (see Figure 6).

#### PV data in the VigiBase^®^

**Figure 7.**
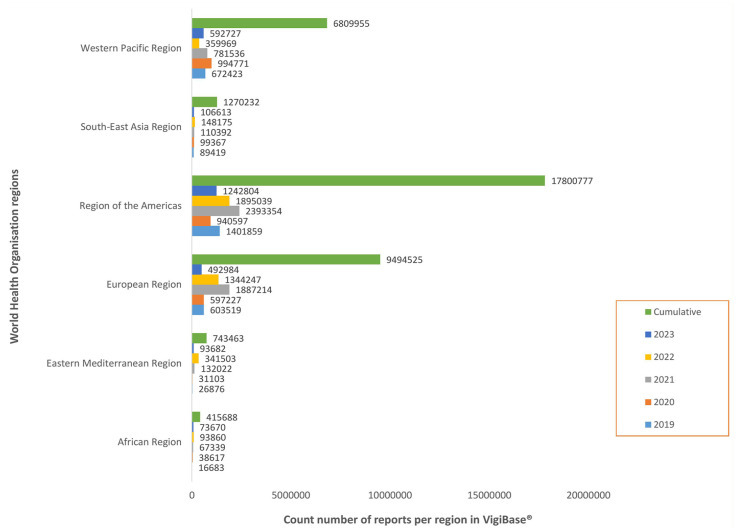
Five-year and cumulative number of individual case safety reports in VigiBase^®^.

The number of ICSR from LMIC in VigiBase^®^ from 2019 to 2023, by WHO Region, is shown in Figure 7. This is a period marked by the introduction of the first malaria vaccine (RTS,S/AS01-Mosquirix) in 2019 and COVID-19 vaccines in 2021. The cumulative number of ICSR by countries was only available for 2022. On 9 August 2022, the cumulative number of ICSR from LMIC in VigiBase^®^ was 463,052 (1.4%) of a global total of 32,234,744, with about two-thirds, 303,196 (0.9%) originating from the African Region. Some of the countries with more advanced PV processes identified in this research were among those accounting for over 60% of total ICSR from the African Region, for example, Nigeria (11.5%), Eritrea (7.9%), United Republic of Tanzania (7.8%), DRC (6.4%) and Ghana (6.2%).

The cumulative number of ICSR from WHO regions on 18 October 2023 is displayed in Figure 7, with the lowest numbers observed in the African Region (415,688 (1%) of a global total of 36,534,640). There is a significant increase from 2020, with a peak in 2021 and 2022, consistent with peaks in other world regions (except the Western Pacific Region), corresponding to the introduction of COVID-19 vaccines.

#### Signal detection and benefit-risk evaluation

Sixty-seven percent (6 out of 9) of interview informants reported that routine data analysis and signal detection were performed in their countries. One representative from an LMIC indicated: ‘*There is a signal review panel with 5 physicians. Most signals are published*’ (NPVC representative, LMIC). However, in most of these countries evaluation of safety signals and benefit–risk assessment leading to regulatory action were not often done systematically.

Findings from the quantitative research showed that 22 respondents (44%) did not know how often signal detection was conducted and 9 (18%) reported that signal detection was not done.

#### Safety-focused regulatory actions

All participants indicated that the NRA in their countries have taken at least one regulatory action for safety reasons in the past such as product recalls, safety alerts, advisory letters, press releases, direct healthcare professional communication or advisory letters and drug withdrawals: ‘*Many regulatory safety actions have been taken within the country, with about 75 products recalled on the basis of PV data*’ (NRA representative, LMIC). However, the majority of safety-driven regulatory actions originated from developed countries rather than from local PV data.

## Discussion

Benefit-risk assessment is an integral part of NRA’s review of authorised medications to ensure that the benefits continue to outweigh the risks. The four themes identified in this research are proposed as transformative steps to strengthen PV data capacity in LMIC.

### Strengthen existing capacity for analysis of national PV data rather than create new or parallel mechanisms

Findings from this research indicated that questions from PV stakeholders on the outcomes of AE collected were one of the factors that triggered the analysis of PV data in LMIC. Initial initiatives that supported PV development in LMIC were mainly focused on raising PV awareness and increasing reporting of ADR.^14^ Consequently, most LMIC have established mechanisms for reporting and collection of AE, resulting in an increase in reports from LMIC. This is an important improvement because underreporting is a global issue and a major challenge in LMIC, especially the African Region. For Africa, the number of ICSR has increased from 103,499 of 11,824,804 (0.88%) in September 2015^30^ to 415,688 of 36,534,640 (1.1%) in October 2023. Overall, our study findings demonstrate that data analysis was not routinely done because of a lack of procedures and tools, poor data quality, insufficient national coordination of PV activities and a limited number of experienced personnel. Core PV outcome indicators such as the number of safety signals detected in the past five years or actions taken recently based on local safety data^31^ were present only in a few countries participating in this research. These findings are consistent with recent assessments of PV systems in LMIC which showed that activities that are essential for benefit-risk assessments such as signal detection were not systematically performed in many LMIC, due to a lack of standardised procedures, structures and dedicated PV budget.^11^

What these findings indicate is the slow progress in PV data analysis and interpretation in LMIC because they mirror the situation reported over 10 years ago.^24,32,33^ Considerable efforts are still required to bring these PV systems to global standards, with the full capacity and ability necessary to identify and tackle medicine safety issues. Capacity for PV data analysis can be strengthened by harnessing existing PV structures and human resources available in-country or within the region or through global partners, preferably through a holistic approach targeting key components of the PV system that are relevant for data analysis.^34,35^ Such an approach will reduce duplicity and increase synergy and impact. These include continuous PV training through training programmes offered by technical agencies^34,36^; increasing transnational collaboration and information exchange^37^; regulatory strengthening partnerships and twinning of regulatory agencies to gain practical knowledge on regulatory activities, such as that established by Swissmedic^38^; and allocating adequate continuous funding to sustain staff and the functions of the safety committees.

Findings from this research showed that the more complex the PV processes, the less they were implemented in participating countries. Whereas mechanisms for AE collection and causality assessment were in place in all countries, procedures for signal detection, RMP and PSUR and benefit-risk assessment were reported by 27 (54%), 21 (42%) and 14 (28%), respectively, of survey respondents. Because these critical PV activities enable the generation of new information about marketed medicines and ensure continuous benefit–risk evaluation, it is imperative that these processes are described and established, and thus applicable in LMIC, particularly where new regulated medical products are introduced. Implementing these procedures implies setting up adequate IT infrastructure and a quality framework to ensure and sustain the functionality of systems and processes. LMIC can implement efficient SOP by adapting processes established in developed countries; making use of available platforms that provide IT solutions for signal detection and management such as VigiLyze^®39^ and ODK; and engaging in regional initiatives that build capacity in signal detection, such as the African Union Smart Safety Surveillance (AU-3S) programme and the South-East Asia Regulatory Network.^40^ In addition, LMIC must also strive to catch up and then stay abreast with new technologies in PV such as ‘big data’ analytics, where advanced analytical techniques are used to extract safety information from large databases,^41–43^ and for applying artificial intelligence and machine learning to PV, with the potential to process and analyse data on an unprecedented scale and speed.^44,45^

One of the minimum requirements of a functional PV system is the availability of a national safety or PV advisory committee that can provide technical assistance on causality assessment, risk assessment and management, case investigation, and, where necessary, crisis management including crisis communication.^46,47^ Beyond national committees are global committees that provide advice and recommendations on the safety of medicines, such as the WHO Advisory Committee on Safety of Medicinal Products^48^ and the EU Pharmacovigilance Risk Assessment Committee.^49,50^ In almost 90% of countries participating in this research, there was at least one national safety committee, reportedly with adequate knowledge and expertise. The major challenge was regularly convening meetings because this often required considerable finance and logistics. Quarterly meetings of safety committees may have been sufficient in the past for nascent PV systems with few suspected AE, but with an increase in reports and with the introduction of innovative products, functional safety committees that meet more regularly are warranted. Concerning the responsibility to review PV data that was not assessed by the safety committee, over 50% of survey respondents did not know or did not provide an opinion. Interestingly, only one respondent mentioned the responsibility of MAH for data analysis. This may indicate a lack of knowledge either about this critical activity or about the process by key PV staff or stakeholders. Reliance on safety committees for causality assessments and review of national PV data has slowed down the acquisition of expertise in data analysis by national PV staff, resulting in a considerable proportion of PV data remaining unexplored. Adequately trained PV staff can contribute to the assessment of non-SAE and less complex SAE and AESI and can carry out signal detection, periodic cumulative data analysis and conduct benefit-risk assessment. The safety committees can then be solicited as an advisory board that supports safety monitoring, evaluating and decision-making tasks as seen in developed countries, thereby releasing funds to address other PV priorities. Assigning PV staff to routine data analysis necessitates appropriate structures, procedures and tools and continuous PV training, including staying abreast with changes in global PV regulatory requirements, as indicated above.

### Implement mechanisms that enable benefit-risk evaluation and decision-making

Another minimum criteria of a functional PV system is the availability of a validated national PV database without which it is impossible to a collate and analyse PV data. All participating countries had a national safety database, with VigiBase^®^ and ODK the most frequently used. Findings showed that the number of ICSR in the safety databases ranged from less than 500 to more than 5000, which was to a large extent consistent with the August 2022 data from VigiBase^®^. Based on few PV processes and outcomes reported in this study, one could infer that the increase in AE reporting was not matched by a corresponding increase in analysis, implying that a large proportion of the data collected is not analysed. For instance, 50% of survey respondents did not know the proportion of AE assessed, while over 40% did not know how often signal detection was conducted. On the other hand, over 60% key interview informants indicated that signal detection was conducted in their countries. This divergence in opinion could suggest that survey respondents’ knowledge of PV data handling and assessment was low, either because such processes did not exist or respondents were not directly involved in these activities.

Less than 30% of study participating countries had mechanisms for the evaluation of safety signals and benefit-risk assessment. In most instances, it was difficult to describe how the outcomes of PV activities were translated into regulatory decisions and actions. Regulatory actions taken in many LMICs did not often result from an accurate analysis of available local PV data but rather mirrored actions performed in developed countries. This approach is not ideal in situations where genetic and environmental factors play a role. For instance, genetic variations in CYP2C19 (the enzyme that metabolises the prodrug clopidogrel, to its active metabolite that inhibits platelet aggregation) are associated with an increased risk of recurrent stroke in patients who are intermediate and poor metabolisers of CYP2C19.^51,52^ Based on the available evidence, the product information in the EU was updated to include this important risk.^53^ This example demonstrates that benefit-risk evaluation and PV decision-making are the main outcomes of PV data analysis, which can only be achieved if supporting related activities are efficiently performed. Although locally generated PV data in LMIC is not yet systematically used for decision-making, there has been some progress in recent years with increasing published safety signals from LMIC, for example, artesunate/amodiaquine-induced extrapyramidal reactions in children and younger adults in Eritrea^54^ and dolutegravir-associated hyperglycaemia in patients with HIV in Uganda.^55^ Nevertheless, achieving effective benefit-risk evaluation necessitates gaining an understanding of the process, assigning tasks for stakeholders (e.g. MAH, NRA, NPVC, safety committees) and defining the steps to translate collected data to decisions and regulatory actions.^56,57^ Many LMIC are still far from this level of organisation and expertise.

Although all proposed actions are important, ensuring the availability of qualified and experienced PV staff, who can routinely analyse national PV data in tandem with national safety committees is a key step. With the appropriate support and structures, staff experienced in PV practice and regulatory guidelines can build on existing structures to develop and implement standardised procedures and mechanisms for correct and efficient analysis of data, and for continuous benefit-risk evaluation. Practical steps to mitigate the broader core challenges of PV in LMIC are described in another submitted manuscript.^58^

### Limitations of the research

The sampling of the qualitative study was purposive and, therefore potentially subject to bias. Only informants with a good understanding of PV systems and procedures, according to the main author’s judgement, were invited to participate. A majority of participants from LMIC were from SSA; therefore, results may largely reflect the situation in the WHO African Region. Lastly, the difference in participants’ PV-related experience between qualitative and quantitative research participants may explain some divergences in opinions seen in this study.

## Conclusion

Findings from this research show that many LMIC have now implemented procedures for reporting and collection of AE and causality assessment, but other key procedures that support the generation of new data such as signal detection data and risk management planning are still missing. A significant amount of data collected is not analysed due to a lack of knowledge, structures, procedures and logistics that support the analysis of data. Consequently, the locally generated PV data are not used to inform safety regulatory actions. Implementing the four themes identified in this research, that is, reinforcing existing PV structures and capacity, developing national PV staff’s knowledge and practice of data analysis, and implementing efficient procedures for data analysis and benefit-risk evaluation will equip LMIC for the analysis of data to support consistent decision-making in PV.

## Supplemental Material

sj-docx-1-taw-10.1177_20420986241300006 – Supplemental material for Pharmacovigilance processes in low- and middle-income countries: moving from data collection to data analysis and interpretation Supplemental material, sj-docx-1-taw-10.1177_20420986241300006 for Pharmacovigilance processes in low- and middle-income countries: moving from data collection to data analysis and interpretation by Olga Menang, Peter van Eeuwijk, Karen Maigetter, Andy Stergachis and Christian Burri in Therapeutic Advances in Drug Safety

sj-docx-2-taw-10.1177_20420986241300006 – Supplemental material for Pharmacovigilance processes in low- and middle-income countries: moving from data collection to data analysis and interpretation Supplemental material, sj-docx-2-taw-10.1177_20420986241300006 for Pharmacovigilance processes in low- and middle-income countries: moving from data collection to data analysis and interpretation by Olga Menang, Peter van Eeuwijk, Karen Maigetter, Andy Stergachis and Christian Burri in Therapeutic Advances in Drug Safety

sj-docx-3-taw-10.1177_20420986241300006 – Supplemental material for Pharmacovigilance processes in low- and middle-income countries: moving from data collection to data analysis and interpretation Supplemental material, sj-docx-3-taw-10.1177_20420986241300006 for Pharmacovigilance processes in low- and middle-income countries: moving from data collection to data analysis and interpretation by Olga Menang, Peter van Eeuwijk, Karen Maigetter, Andy Stergachis and Christian Burri in Therapeutic Advances in Drug Safety

sj-docx-5-taw-10.1177_20420986241300006 – Supplemental material for Pharmacovigilance processes in low- and middle-income countries: moving from data collection to data analysis and interpretation Supplemental material, sj-docx-5-taw-10.1177_20420986241300006 for Pharmacovigilance processes in low- and middle-income countries: moving from data collection to data analysis and interpretation by Olga Menang, Peter van Eeuwijk, Karen Maigetter, Andy Stergachis and Christian Burri in Therapeutic Advances in Drug Safety

sj-docx-6-taw-10.1177_20420986241300006 – Supplemental material for Pharmacovigilance processes in low- and middle-income countries: moving from data collection to data analysis and interpretation Supplemental material, sj-docx-6-taw-10.1177_20420986241300006 for Pharmacovigilance processes in low- and middle-income countries: moving from data collection to data analysis and interpretation by Olga Menang, Peter van Eeuwijk, Karen Maigetter, Andy Stergachis and Christian Burri in Therapeutic Advances in Drug Safety

sj-xlsx-4-taw-10.1177_20420986241300006 – Supplemental material for Pharmacovigilance processes in low- and middle-income countries: moving from data collection to data analysis and interpretation Supplemental material, sj-xlsx-4-taw-10.1177_20420986241300006 for Pharmacovigilance processes in low- and middle-income countries: moving from data collection to data analysis and interpretation by Olga Menang, Peter van Eeuwijk, Karen Maigetter, Andy Stergachis and Christian Burri in Therapeutic Advances in Drug Safety

## Declarations

**Ethics approval and consent to participate:** This study was approved by the research commission of the Swiss Tropical and Public Health Institute (Swiss TPH, Allschwil, Switzerland). The Ethics Committee of Northwestern and Central Switzerland (EKNZ) waived the need for formal ethics approval (Req-2020-00232) since it does not fall under the Swiss Human Subject Research Act. The EKNZ reviewed the submitted documents and confirmed that the research project fulfilled the general ethical and scientific standards for research with humans according to Art 51 of the Swiss Human Subject Research Act. Consent to participate: At the start of the interview, qualitative research participants verbally consented to participate in the study and to record the interview. Survey participants agreed that by filling out the questionnaire they were consenting to participate in the study.

**Consent for publication:** At the start of the interview, qualitative research participants verbally consented to the use of the information provided in the research for related publications. By filling out the online questionnaire, survey participants consented to the anonymised use of information provided in the study for related publications.

**Availability of data and materials:** All data generated or analysed during this study are included in this published article (and its Supplemental Information Files).
