# Supplementary material for: Pharmacovigilance processes in low- and middle-income countries: moving from data collection to data analysis and interpretation
Source: Ther Adv Drug Saf. 2025 Jun 11;16:20420986241300006. doi: 10.1177/20420986241300006 (PMC12159475; doi:10.1177/20420986241300006)
Supplement: sj-docx-1-taw-10.1177_20420986241300006 – Supplemental material for Pharmacovigilance processes in low- and middle-income countries: moving from data collection to data analysis and interpretation [file sj-docx-1-taw-10.1177_20420986241300006.docx]

**Supplemental File 1**

| Interview Guide for qualitative research  Pharmacovigilance processes in low- and middle-income countries: moving from data collection to data analysis and interpretation |
| --- |
| Welcome and introduction |
| - Introduction of interviewer - Provide the background for the interview and an overview of the PhD research - Express gratitude to the participant for agreeing to contribute to the research - Provide details on the length of the interview - Obtain verbal consent to record interview and include information provided in the research |
| Part 1: Respondent’s role in the national pharmacovigilance system |
| 1. What organization do you work for? 2. What position do you hold within your organization? 3. What are your roles and responsibilities with regards to the national pharmacovigilance (PV) system? 4. Would you say you have any influence on the decisions taken on the functionality of the national PV system? What influence and why so? |
| Part 2: Evolution of national pharmacovigilance systems: from data collection to data analysis and interpretation |
| 1. What are the events that have boosted safety data analysis in the past years? Why so? 2. In your opinion, would you say the capacity for analysis of national safety data from all sources (including vaccines and drugs) is adequate? 3. Who is responsible for data analysis at the national level? 4. Do the national guidelines provide guidance for data analysis? 5. Are there standard operating procedures (SOP) in place to ensure adequate data collection and analysis? Which ones? 6. What is the role of the national safety expert review committee? 7. In your opinion is the national safety review committee sufficiently empowered to analyze PV data? 8. Who does the assessment of the safety data that is not analyzed by the national safety committee? 9. How often is the national PV data reviewed with a focus on identifying of safety signals? 10. How many safety signals were detected in the past five years? 11. What actions have recently been taken based on analysis of safety data? 12. What is the source of data for regulatory decisions? 13. What are the gaps in data analysis and what steps can be taken to address these gaps? |
| Closing |
| - Is there anything else you would like to add? - Do you have any questions about the interview, the research and the researcher? |
| Thank you for your participation and contribution to this research! |
