# Supplementary material for: Pharmacovigilance processes in low- and middle-income countries: moving from data collection to data analysis and interpretation
Source: Ther Adv Drug Saf. 2025 Jun 11;16:20420986241300006. doi: 10.1177/20420986241300006 (PMC12159475; doi:10.1177/20420986241300006)
Supplement: sj-docx-2-taw-10.1177_20420986241300006 – Supplemental material for Pharmacovigilance processes in low- and middle-income countries: moving from data collection to data analysis and interpretation [file sj-docx-2-taw-10.1177_20420986241300006.docx]

**Supplemental File 2**

| Questionnaire for online survey (Open Data Kit Collect)  Pharmacovigilance processes in low- and middle-income countries: moving from data collection to data analysis and interpretation |
| --- |
| Welcome and introduction |
| Thank you for participating in this survey on evolution of national pharmacovigilance (PV) systems in low- and middle-income countries (LMIC). This survey aims to understand the capacity of data analysis in LMIC and how systems have evolved from merely collecting adverse drug reactions to analysing and interpreting this data and using the data to inform PV decisions in LMIC. Your contribution to this research will provide valuable information on optimal strategies to build more functional and resilient PV systems in LMIC. Your participation in this online survey is voluntary and anonymous, and your responses will be confidential. We will not collect identifying information such as your name, email address or IP address. By completing this questionnaire you are consenting to participate in the study and consenting to use the information provided in the research. Completing this this online survey will take approximately 30 minutes. |
| Part A: Respondent’s role in the national pharmacovigilance system |
| 1. What organization do you work for?*   National Regulatory Authority (NRA)  PV centre  National Immunisation Programme (NIP)  Donor agencies  Technical Agency (TA)  Industry  Other __________   1. What position do you hold within your organization? __________ 2. How many years of PV experience do you have?   Less than 1 year  2 to 5 years  6 to 10 years  11 to 15 years  More than 16 years |
| Part B: Evolution of national pharmacovigilance systems: from data collection to analysis and interpretation |
| 1. Are there sufficiently competent staff (i.e., education, training, skills and experience) assigned to perform vigilance activities?   ☐Yes  ☐No  ☐I don’t know   1. In your opinion, is the capacity for analysis of national PV data adequate?   ☐Strongly Disagree  ☐Disagree  ☐Neither agree nor disagree  ☐Agree  ☐Strongly Agree   1. Tick which of the following procedures have been implemented for the collection and management of adverse events in your country:   ☐ Collection of adverse events using a standardized form?  ☐ Reporting mechanism and flow of adverse event reports to the central level?  ☐Data entry (including narrative writing and medical coding) into the national database (or Vigibase™)  ☐Serious adverse event investigation (as applicable)  ☐Causality assessment   1. Tick which of the following procedures have been implemented for periodic analysis of pharmacovigilance data in your country:   ☐Signal detection, evaluation and management  ☐Preparation of periodic safety update reports (PSURs)  ☐ Risk management plans and risk minimization  ☐Benefit risk evaluation  ☐Active safety surveillance   1. Tick the processes for which PV staff are sufficiently trained and have adequate capacity:   ☐ Collection of adverse events using a standardized form?  ☐ Reporting mechanism and flow of adverse event reports to the central level?  ☐Data entry (including narrative writing and medical coding) into the national database (or Vigibase™)  ☐Serious adverse event investigation (as applicable)  ☐Causality assessment  ☐Signal detection, evaluation and management  ☐Preparation of periodic safety update reports (PSURs)  ☐ Risk management plans and risk minimization  ☐Benefit risk evaluation  ☐Active safety surveillance   1. Is there a national safety review committee(s) in your country?   ☐Yes  ☐No  ☐I don’t know   1. If yes, how frequently do they meet?   ☐Monthly  ☐Quarterly  ☐Biannually  ☐Annually  ☐Other __________   1. In your opinion is the national safety review committee sufficiently scientifically empowered to analyze PV data?   ☐Yes  ☐No  ☐I don’t know   1. Who does the assessment of the safety data that is not analyzed by the national drug safety committee(s)? __________ 2. What is the frequency of assessment?   ☐Weekly  ☐Monthly  ☐Quarterly  ☐Biannually  ☐Annually  ☐Other   1. What database is used for adverse event* collation and analysis? (*Adverse event here can refer to adverse drug reactions (ADRs) or adverse event following immunization (AEFIs)) __________ 2. How many adverse events are currently in the national safety database (or Vigibase™)?   ☐Less than 500  ☐Less than 1000  ☐More than 1000  ☐More than 2000 but less than 5000  ☐More than 5000  ☐I don’t know   1. What proportion of these adverse events serious adverse events (SAEs)?   ☐Less than 10%  ☐Less than 25%  ☐More than 50%  ☐More than 80%  ☐I don’t know   1. What proportion of SAEs in the database have been analysed?   ☐Less than 10%  ☐Less than 25%  ☐More than 50%  ☐More than 80%?  ☐I don’t’ know   1. What proportion of non-serious cases in the database have been analysed?   ☐Less than 10%  ☐Less than 25%  ☐More than 50%  ☐More than 80%?  ☐I don’t know   1. How often is the national PV data analysed with a focus on detection of safety signals?   ☐Monthly  ☐Quarterly  ☐Biannually  ☐Annually  ☐Other__________   1. What proportion of safety signals have been successfully analysed and closed with appropriate regulatory recommendation or action)?   ☐None  ☐Less than 10%  ☐Less than 25%  ☐More than 50%  ☐More than 80%?  ☐I don’t know   1. Tick which of these regulatory actions have recently been taken based on analysis of PV data in the last year?   ☐None  ☐Product / batch recall  ☐Product withdrawal  ☐Safety alert to the public  ☐Safety communication to HCPs  ☐Update to product information  ☐I don’t know  ☐Other __________ |
| Thank you for your participation and contribution to this research! |
