# Supplementary material for: Pharmacovigilance processes in low- and middle-income countries: moving from data collection to data analysis and interpretation
Source: Ther Adv Drug Saf. 2025 Jun 11;16:20420986241300006. doi: 10.1177/20420986241300006 (PMC12159475; doi:10.1177/20420986241300006)
Supplement: sj-docx-3-taw-10.1177_20420986241300006 – Supplemental material for Pharmacovigilance processes in low- and middle-income countries: moving from data collection to data analysis and interpretation [file sj-docx-3-taw-10.1177_20420986241300006.docx]

**Supplemental File 3**

**Codebook for qualitative research**

**Pharmacovigilance processes in low- and middle-income countries: moving from data collection to data analysis and interpretation**

| Part 1 : Respondent’s role in the national pharmacovigilance (PV) system | | | |
| --- | --- | --- | --- |
| Codes | **Sub codes** | **Code description** | **Assumption/comment** |
| Organisation | Technical and financial partner (TFP) | Where the respondent works | q |
|  | National regulatory authorities (NRA) |  |  |
|  | National PV Centre (NPVC) |  |  |
|  | National Immunization Programs (NIP) |  |  |
|  | Other Public Health Program (PHP) |  |  |
| Position | PV head or lead | Number of years of PV experience | Senior positions can influence PV system functionality |
|  | PV officer |  |  |
| Roles and responsibilities | Lead PV | Have roles and responsibilities vis a vis the PV system | None |
|  | Coordinate PV |  |  |
|  | PV focal point |  |  |
|  | Technical support |  |  |
| Influence on PV decisions | PV decision | Can influence decisions taken on the PV system | Senior positions can drive PV decisions |
|  | PV implementation |  |  |
| Part 2 : Evolution of national pharmacovigilance systems: from data collection to analysis and interpretation | | | |
| Codes | **Sub codes** | **Code description** | **Assumption/comment** |
| Data analysis trigger | Drug introduction | What triggered the need to establish procedures for data analysis | As a systems evolves and data is collected regularly, capacity for data analysis should be built and procedures implemented |
|  | Natural progression |  |  |
|  | Vaccine introduction |  |  |
|  | Adverse events |  |  |
| Data analysis capacity | Adequate data analysis | If informant believes that national safety data is adequately analysed | As systems evolve, these are outcomes of PV performance |
|  | Inadequate data analysis |  |  |
|  | No analysis |  |  |
| Data analysis responsibility | PV staff |  |  |
|  | Safety committee |  |  |
|  | Both |  |  |
| Procedural guidelines | PV staff guidelines | Guidelines that describe PV processes | At the minimum there should be guidelines with instruction on handling PV data |
|  | Industry guidelines |  |  |
| PV SOP | ADR reporting SOP | Availability of SOP that describe critical PV processes | Documented and implemented PV procedures indicate degree of advancement |
|  | PSUR SOP |  |  |
|  | Signal detection SOP |  |  |
|  | Risk management SOP |  |  |
|  | Safety communication SOP |  |  |
|  | No PV SOP |  |  |
|  | Other SOP |  |  |
| National safety committee | Safety committee functional | Availability of safety committees to support data review and assessment | If committee is functional, routine causality assessment and data analysis is ensured |
|  | Safety committee not functional |  |  |
| Role of safety committee | Causality assessment |  |  |
|  | Investigations |  |  |
|  | Data analysis |  |  |
| Experience of safety committee | Established |  |  |
|  | New committees |  |  |
| Assessment of other PV data | PV staff | Assessment of PV data not reviewed by safety committee | If PV staff is sufficiently experienced. they can analyse PV data to support committee |
|  | Regular data analysis |  |  |
|  | Occasionally |  |  |
|  | Not done |  |  |
| Routine signal detection | Exists | Availability of clear guidance for signal detection | Signal detection is the crux of PV |
|  | Does not exist |  |  |
|  | Safety signals identified |  |  |
| Continuous benefit risk evaluation | Qualitative data review | Availability of clear guidance for benefit risk evaluation | The evaluation of the benefit–risk balance is a key element across the entire life cycle of a medicinal product |
|  | Other methods |  |  |
| Regulatory actions | Based on national data | Safety driven regulatory actions based on safety data collected and analysed at the national level | Outcome of data analysis is only possible if data are analysed |
|  | Based on external action |  |  |
|  | Type of actions – Recalls, Withdrawal, Safety alert, Communication to HCPs  Advisories |  |  |
|  | Number of actions |  |  |
| Data analysis gaps | Procedures | Describe the bottlenecks concerning data analysis | Identifying challenges is the first step to resolving them |
|  | Expertise |  |  |
|  | Adequate tools |  |  |
|  | Poor data |  |  |
| Strengthen data analysis | Implemented procedures | Propose ways to build capacity for data analysis | As a systems evolves and data is collected regularly, capacity for data analysis should be built and procedures implemented |
|  | Digitalize process |  |  |
|  | Develop tools |  |  |
|  | Train staff |  |  |
|  | Train safety committees |  |  |
|  | Empower safety committees |  |  |
|  | Improve data quality |  |  |
|  | Increase ADR |  |  |
