# Supplementary material for: Pharmacovigilance processes in low- and middle-income countries: moving from data collection to data analysis and interpretation
Source: Ther Adv Drug Saf. 2025 Jun 11;16:20420986241300006. doi: 10.1177/20420986241300006 (PMC12159475; doi:10.1177/20420986241300006)
Supplement: sj-docx-5-taw-10.1177_20420986241300006 – Supplemental material for Pharmacovigilance processes in low- and middle-income countries: moving from data collection to data analysis and interpretation [file sj-docx-5-taw-10.1177_20420986241300006.docx]

**Supplemental File 5**

**Consolidated criteria for reporting qualitative studies (COREQ): 32-item checklist**

**Manuscript: Pharmacovigilance processes in low- and middle-income countries: moving from data collection to data analysis and interpretation**

| Item No. | Topic | Guide Questions/Description | Reported on Page No. |
| --- | --- | --- | --- |
| Domain 1: Research team and reflexivity | | | |
| *Personal Characteristics* | | | |
| 1 | Interviewer/facilitator | Which author/s conducted the interview or focus group? | Page 8 |
| 2 | Credentials | What were the researcher’s credentials? E.g. PhD, MD | Page 1 |
| 3 | Occupation | What was their occupation at the time of the study? | Page 1 |
| 4 | Gender | Was the researcher male or female? | Page 1 |
| 5 | Experience and training | What experience or training did the researcher have? | Page 1 |
| *Relationship with participants* | | | |
| 6 | Relationship established | Was a relationship established prior to study commencement? | Page 8 |
| 7 | Participant’s knowledge of the interviewer | What did the participants know about the researcher? e.g. personal goals, reasons for doing the research | Page 8, Supplemental File 1 |
| 8 | Interviewer’s characteristics | What characteristics were reported about the inter viewer/facilitator? e.g. bias, assumptions, reasons and interests in the research topic | Supplemental File 1 |
| Domain 2: Study design | | | |
| *Theoretical framework* | | | |
| 9 | Methodological orientation and theory | What methodological orientation was stated to underpin the study? e.g. grounded theory, discourse analysis, ethnography, phenomenology, content analysis | Page 8 |
| *Participant selection* | | | |
| 10 | Sampling | How were participants selected? e.g. purposive, convenience, consecutive, snowball | Page 7 |
| 11 | Method of approach | How were participants approached? e.g. face-to-face, telephone, mail, email | Page 7 |
| 12 | Sample size | How many participants were in the study? | Page 9 |
| 13 | Non-participation | How many people refused to participate or dropped out? Reasons? | Page 9 |
| *Setting* | | | |
| 14 | Setting of data collection | Where was the data collected? e.g. home, clinic, workplace | Page 8 |
| 15 | Presence of non-participants | Was anyone else present besides the participants and researchers? | Page 8 |
| 16 | Description of sample | What are the important characteristics of the sample? e.g. demographic data, date | Pages 9-11 |
| *Data collection* | | | |
| 17 | Interview guide | Were questions, prompts, guides provided by the authors? Was it pilot-tested? | Page 8 |
| 18 | Repeat interviews | Were repeat interviews carried out? If yes, how many? | NA |
| 19 | Audio/visual recording | Did the research use audio or visual recordings to collect the data? | Page 8 |
| 20 | Field notes | Were ﬁeld notes made during and/or after the interviews or focus groups? | NA |
| 21 | Duration | What was the duration of the interviews or focus groups? | Page 8 |
| 22 | Data saturation | Was data saturation discussed? | Page 7 |
| 23 | Transcripts returned | Were transcripts returned to participants for comment and/or correction? | NA |
| Domain 3: Analysis and ﬁndings | | | |
| *Data analysis* | | | |
| 24 | Number of datacoders | How many datacoders coded the data? | Page 8 |
| 25 | Description of the coding tree | Did authors provide a description of the coding tree? | Pages 8 Supplemental File 3 |
| 26 | Derivation of themes | Were themes identiﬁed in advance or derived from the data? | Pages 8 Supplemental File 3 |
| 27 | Software | What software, if applicable, was used to manage the data? | NA |
| 28 | Participant checking | Did participants provide feedback on the ﬁndings? | NA |
| *Reporting* | | | |
| 29 | Quotations presented | Were participant quotations presented to illustrate the themes/ﬁndings? Was each quotation identiﬁed? e.g. participant number | Pages 13 to 17 |
| 30 | Data and ﬁndings consistent | Was there consistency between the data presented and the ﬁndings? | Pages 13 to 17 |
| 31 | Clarity of major themes | Were major themes clearly presented in the ﬁndings? | Pages 12 to 17 |
| 32 | Clarity of minor themes | Is there a description of diverse cases or discussion of minor themes? | Page 12 to 17 |

*Developed from: Tong A, Sainsbury P, Craig J. Consolidated criteria for reporting qualitative research (COREQ): a 32-item checklist for interviews and focus groups. International Journal for Quality in Health Care. 2007. Volume 19, Number 6: pp. 349 – 357*
