# Supplementary material for: Pharmacovigilance processes in low- and middle-income countries: moving from data collection to data analysis and interpretation
Source: Ther Adv Drug Saf. 2025 Jun 11;16:20420986241300006. doi: 10.1177/20420986241300006 (PMC12159475; doi:10.1177/20420986241300006)
Supplement: sj-docx-6-taw-10.1177_20420986241300006 – Supplemental material for Pharmacovigilance processes in low- and middle-income countries: moving from data collection to data analysis and interpretation [file sj-docx-6-taw-10.1177_20420986241300006.docx]

**Title and description for all supplemental material items supplied**

**Supplemental File 1**

**Title:** Interview Guide for qualitative research

**Description:** The interview guide was developed to guide the in-depth interviews and gain profound and practical understanding of how PV systems in high-, middle- and low-income countries are established and how they are sustained.

**Supplemental File 2**

**Title:** Questionnaire for online survey

**Description:** Quantitative data were collected using a standardised questionnaire which was set up in Open Data Kit Collect (ODK - https://opendatakit.org)

**Supplemental File 3**

**Title:** Codebook for qualitative research

**Description:** The respondents’ statements from the interviews were transcribed verbatim by the first author and deductive coding was used to code the transcript. For this purpose, a codebook was developed. The codebook outlined the categories, codes, and subcodes with the corresponding description of the codes and assumptions or rationales for collecting the data.

**Supplemental File 4**

**Title:** Qualitative framework matrix

**Description:** Spreadsheet to summarize and ‘chart’ each transcript into a matrix consisting of categories, codes and subcodes.

To prevent the possibility of identifying the respondent through their job title and the organisation for which they worked, the respondents’ countries, organisations and job titles are not included in the framework matric. Any responses that could provide a hint for possibly identifying the respondents were edited such that the text is completely anonymised.

**Supplemental File 5**

**Title:** Consolidated criteria for reporting qualitative studies (COREQ): 32-item checklist

**Description:** This is a checklist for explicit and comprehensive reporting of the qualitative research
